# MicroRNAs utilization as effective factors on hematopoietic stem cell transplantation, its outcomes and prognosis; a comprehensive systematic review

**DOI:** 10.1186/s12885-024-12640-9

**Published:** 2024-07-24

**Authors:** Negar Habibollahzadeh, Samin Yavari, Yasin Mirazimi, Amir Hossein Aghayan, Atefeh Davoudian, Mohammad Rafiee

**Affiliations:** 1 Student Research Committee, Department of Medical Laboratory Sciences, School of Paramedical Sciences, Zanjan University of Medical Sciences, Zanjan, Iran; 2 Deputy of Research and Technology, Zanjan University of Medical sciences, Zanjan, Iran; 3 Department of Medical Laboratory Sciences, School of Paramedical Sciences, Zanjan University of Medical Sciences, Zanjan, Iran

**Keywords:** MicroRNA, Hematopoietic stem cell transplantation, Outcomes, Survival, Systematic review

## Abstract

**Introduction:**

The therapeutic method for many malignant and non-malignant diseases is hematopoietic stem cell transplantation (HSCT), but it is not always fully successful in all patients. Indeed, HSCT can be influenced by a variety of factors. Here we reviewed the effect of microRNAs (miRs) on HSCT-related outcomes, like survival, infections, relapse, engraftment, and so on, systematically.

**Method:**

WOS, Scopus, PubMed, Google Scholar, and ProQuest databases were searched. The PRISMA guideline was performed, and 24 studies were included through quality assessment. Classified data extraction was done based on the type of disease.

**Results:**

The systematic review identified 47 miRs effective on HSCT. The role of miRs as tumor suppressors or oncogenes is reported in acute myeloblastic and lymphoblastic leukemia patients undergoing HSCT due to their effects on overall or event-free survival. Additionally, relapse after HSCT in multiple myeloma is correlated with miRs expression. Also, recovery from post-autologous HSCT cytopenia or platelet and neutrophil engraftment can be influenced by miRs. We highlighted here reports on specific miRs.

**Conclusion:**

We reported prognostic miRs for in-depth clinical management of the HSCT process and its outcomes. Also, miRs are introduced for the prevention of HSCT-related complications, and future studies are suggested to evaluate personalized medicine’s utilization of miRs in therapeutic methods like HSCT in neoplasia.

## Introduction

Hematopoietic stem cell transplantation (HSCT) is a process that starts with short periods of high-dose chemotherapy, radiotherapy, or both, followed by the infusion of healthy stem cells to replace unhealthy bone marrow cells [1]. HSCT is a significant step for treating bone marrow tumors and compensating for inefficiency in patients with hematologic and non-hematologic disorders or malignancies. HSCT indications are: (1) malignancies like multiple myeloma (MM), Hodgkin’s and non-Hodgkin’s lymphoma, acute myeloid leukemia (AML), acute lymphoid leukemia (ALL), myelodysplasia, chronic myeloid leukemia (CML), and chronic lymphoid leukemia (CLL), (2) non-malignancies like aplastic anemia, severe combined immunodeficiency, thalassemia, and sickle cell anemia [2]. The microenvironment of HSCs in bone marrow or signals from the surrounding niche affect the fate of HSCT and its complications, like microRNAs (miRs) [3]. MiRs are a large class of short non-coding RNAs that may affect cell proliferation, differentiation, apoptosis, and tumorigenesis. The expression of miRNAs and related processing proteins is important for the maintenance of hematopoietic stem cells [4]. MiRs could play a dual role in regulating the development of cancers such as AML and ALL, acting as both oncogenes and tumor suppressor genes [5]. In previous studies, it was hypothesized that the abnormal expression of miRs set the stage for the development of post-HSCT morbidities such as GVHD, infection, and organ failures, as demonstrated in cause of death (COD), regardless of baseline variables such as characteristics of primary cancer or transplant type [6]. As in the study of Lin Fu et al., it has been indicated that in the group with high expression of miR-338, patients with AML receiving allogeneic hematopoietic stem cell transplantation (allo-HSCT) had longer overall survival (OS) and event-free survival (EFS) than those receiving chemotherapy only [5]. MM patients with lower levels of miR-19b or miR-331 had shorter progression-free survival (PFS) than those with higher levels, and those with low levels of both miRs had shorter PFS than those with high levels of either miRs [7]. In addition, it has been indicated that high expression of miR-98 is related to a good prognostic factor in AML patients who only received chemotherapy, whereas patients with low expression of miR-98 may benefit from allo-HSCT [8]. Also, it is revealed that higher levels of miR-15a, miR-16, miR-126, and miR-146a on day 0 correlated with a longer time to engraftment [9]. So, in this study, we aimed to review systematically the role of miRs in HSCT and the factors associated with it, including mobilization, conditioning regimens, and engraftment. Other HSCT-related complications, such as engraftment, relapse, survival rate, and so on, are reviewed as well.

## Methods

### Eligibility criteria

We performed a systematic review, registered on PROSPERO (ID: CRD42022341456). This systematic review was carried out based on the guidelines of the Preferred Reporting Items for Systematic Reviews and Meta-Analyses (PRISMA) statement. According to the Prospero protocol, the extracted articles were reviewed to determine their eligibility criteria. The inclusion criteria were: studies that investigated the role of miRs (including cellular, circulatory, and exosomal) in autologous and allogeneic HSCT-related processes (including mobilization and conditioning chemotherapy) and outcomes (including homing, exhaustion, and engraftment of HSCs, blood cell recovery after transplantation, mortality, survival, remission, and relapse). The exclusion criteria were: (A) studies with no permission to fully access them; (B) duplicate articles; (C) studies with a lack of data; (D) studies that only use an in-silico approach; (E) gray literature with unavailable or insufficient data; (F) studies that were not in English; and (G) studies that worked on non-human samples.

### Information sources

According to PRISMA Statement [10], the WOS, Scopus, PubMed, Google Scholar, and ProQuest databases were searched for articles published through December 2023 on the association between the role of miRs and HSCT.

### Search Strategy

In order to not miss the studies, MESH words were used. MeSH and non-MeSH keywords were used to find related studies: #1 “MicroRNAs”, #2 “Hematopoietic Stem Cell Transplantation”, #3 “Peripheral Blood Stem Cell Transplantation”, #4 “Cord Blood Stem Cell Transplantation”, #5 “Hematopoietic Stem Cell Mobilization”, #6 “Stem Cell Transplantation”, #7 “Transplantation, Heterologous”, #8 “Mortality”, #9 “Remission Induction”; #10 “Induction Chemotherapy”, #11 “Recurrence”, #12 “Transplantation Conditioning”, #13 “Engraftment”, #14 “Survival” and #15 “Homing”. The combination of search keywords was as follows: #1 AND (#2 OR #3 OR #4 OR #5 OR #6 OR #7) AND (#8 OR #9 OR #10 OR #11 OR #12 OR #13 OR #14 OR #15).

### Selection process

After removing duplicate studies, based on inclusion criteria and exclusion criteria, the titles and abstracts of all extracted articles from databases were reviewed by two researchers (YM and AHA). If there was any disagreement, it was resolved by discussion, but if the disagreement still remained, the final decision was made by the third researcher (MR). Initial screening of the extracted studies was carried out using the web-based software Rayyan. The bias risk assessment was carried out using the Newcastle-Ottawa Scale (NOS) criteria.

### Data extraction and quality assessment

Two reviewers (NH and SY) independently extracted data and classified it in separate Excels. The two excels mentioned were compared and consulted by a third reviewer (MR). The quality control was performed by two reviewers (NH and SY) and finalized by a third person (MR). Considering all studies were cohorts, the selection, comparability, and outcomes were carefully reviewed.

### Data items

As for each included article, these traits based on pre-specified Excel were considered: the first author’s name; the name of the miRs; the year; the country; the detection methods; the sample type; the sample size of patients; the sample size of control; the type of disease of the patient who received transplantation; the type of transplantation; the relationship between miRs and the disease (survival, engraftment, mobilization); the expression of miRs (up or down regulation); the follow-up duration; the statistical parameters (sensitivity, specificity, AUC, HR, OR, OS, and so on).

## Results

### Study selection

**Fig. 1:**
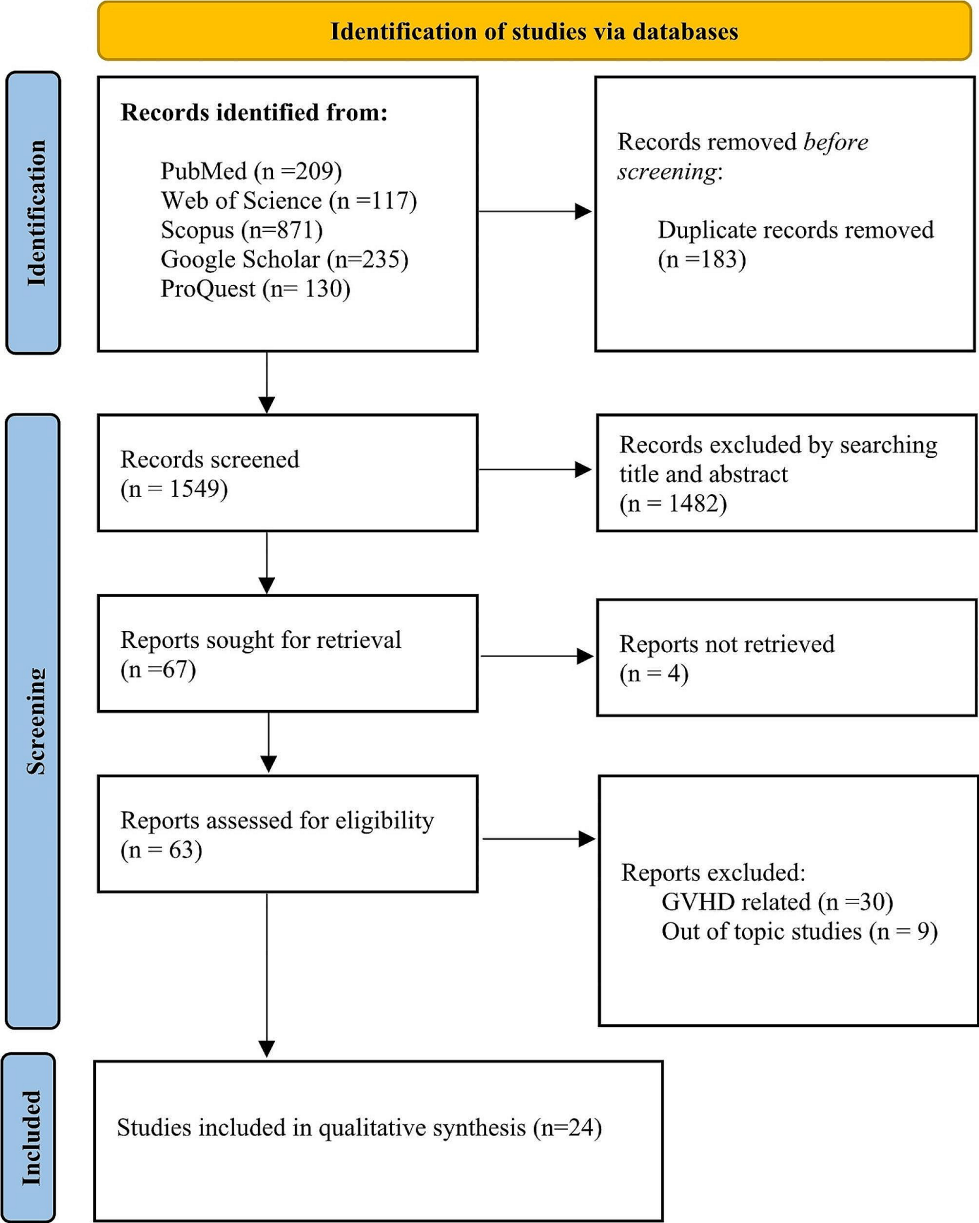
PRISMA flow diagram

Based on the PRISMA flow diagram [10], the study selection process is shown in Fig. 1. A total of 1732 studies were extracted from the mentioned database. Initially, 183 duplicate articles were removed. After two researchers initially screened 1549 titles and abstracts, 1482 were excluded because they were not compatible with the inclusion and exclusion criteria. Next, 67 studies were selected for full-text examination. 4 full-text studies could not be retrieved, and 38 studies were excluded as a result of the reasons outlined in Fig. 1. Finally, the number of articles included in the qualitative synthesis was 24 [3–9, 11–27]. The NOS bias risk assessment for the included article is shown in Table 1.

**Table 1:** Supplemental Content, which illustrates study quality assessed via the Newcastle-Ottawa Scale checklist

Study	Selection	Comparability	Outcome	Total score
Vanesa MartınPalanco 2010	****	*	**	7
Xiao-Peng Tian 2019	****	**	*	7
Chen Yang 2018	****	**	**	8
GAOQI ZHANG 2019	****	**	**	8
Huihui Zhang 2019	****	**	*	7
JILEI ZHANG 2019	****	**	***	9
Jing-dong Zhou 2019	****	**	**	8
Katja Seipel 2020	****	**	**	8
Lin Fu 2019	****	**	***	9
Mingshan Niu 2019	****	**	**	8
Ning Hu 2019	****	**	**	8
Shi Jinlong 2015	****	**	**	8
Ting-Juan Zhang 2020	****	**	**	8
Zhiheng Cheng 2017	****	**	***	9
Zhiheng Cheng 2021	****	**	***	9
Gretchen A 2017	****	**	**	8
Mateusz Nowicki 2016	****	**	*	7
Mateusz Nowicki 2016	****	*	*	6
Mohamed L. Sorror 2018	****	**	*	7
Mohammad Rafiee 2021	****	*	*	6
Peter L. Pontoppidan 2014	****	*	*	6
Alfons Navarro 2015	****	*	*	6
de Larrea 2012	****	*	**	7
Sung-Soo Park 2019	****	*	*	6

### Study characteristics

We reviewed 24 studies conducted between 2012 and 2023. Most studies focused on a specific miR and its effects following HSCT and chemotherapy. In some studies, patients were divided into two groups based on their treatment method and observed for high and low expression of miRs, which were then compared. Also, we classified the articles according to the patient cohorts studied, resulting in four groups: acute myeloid leukemia (AML), acute lymphoblastic leukemia (ALL), multiple myeloma (MM), and a general group.

### Results of syntheses

Our analysis focused on the impact of miR expression levels on patients who underwent HSCT or received chemotherapy within each of the four aforementioned groups. Specifically, we examined the association between high or low miR expression and clinical outcomes (OS and EFS) in these patient populations.

#### Patients with AML

There are studies on the comparison of chemotherapy and HSCT according to miRs expression (Table 2). In the patients receiving chemotherapy, high expression of miR-425, miR − 98, and miR − 25 was associated with higher OS and EFS, but in allo-HSCT groups, no significant differences were seen between high expression groups and low expression groups [8, 11, 18, 26]. AML patients going under allo-HSCT concluded that high expression of miR-500 can result in shorter OS and EFS [4]. Studies examining miR-20b, miR-338, and miR-363 have figured out that high expression of these miRs in AML patients undergoing chemotherapy is associated with longer OS and EFS. For patients undergoing allo-HSCT, although no significant differences were observed between the high and low expression groups. Allo-HSCT may still overcome the negative impact of high miR expression. Conversely, high expression of miR-99a in patients undergoing allo-HSCT can reduce the OS and EFS and unfortunately, in these patients, allo-HSCT may not overcome the negative impact of miR-99a expression [5, 12, 13, 25]. The combination of high expression of let-7a-2-3p and low expression of miR-188-5p can significantly impact the OS, EFS, and relapse duration in CN-AML (cytogenetically normal AML) patients. Furthermore, each of these factors on its own can also lead to longer OS and EFS whether they receive HSCT or not [23]. Also, low expression of the *BCL2* gene may lead to better OS and leukemia-free survival (LFS) in patients undergoing allo-HSCT. *BCL2* expression was found to have a negative correlation with tumor suppressive miRs, such as miR-195, miR-497, and miR-193b, which could potentially explain the outcomes of low expression of *BCL2* [16]. Similarly, low expression of *DNMT3A* was associated with longer OS and LFS in patients undergoing allo-HSCT. MiR-429 and miR-29b are found to directly regulate the expression of the protein DNMT3A in AML cells. These findings suggest that miRs may play a role in the regulation of *DNMT3A* expression and its effect on patients [27]. Also, AML patients with increased expression of the *MN1* or *FoxP1* genes at the time of diagnosis had a significantly shorter PFS and OS after intensive induction chemotherapy and autologous HSCT (AHSCT). Interestingly, elevated expression of miR-181a-5p, a putative tumor suppressive microRNA, was found to be predictive of a positive outcome, in contrast to *MN1* and *FoxP1*. Analysis of non-coding RNAs revealed an inverse correlation between *MN1* and miR-20a-5p and miR-181b-5p expression. These results suggest that *MN1*, *FoxP1*, and miR-181a-5p could serve as prognostic markers in AML patients who undergo intensive induction chemotherapy and ASCT [22].

**Table 2:** Extracted data from AML related studies with miRs assessment in HSCT

Study	MiRs	Method	Sample type	No. Sample	Age and Sex of samples	HSCT type	Role of miRs	Follow up parameter	Follow up*	Survival parameters	% Of relapse	Other measured parameters	Main finding or purpose
Chen Yang 2018 [11]	miR-425	qRT-PCR, Sequencing/ In silico		162	Age: above 60 years: 81 and under 60 years: 81 71 females and 91 males	Allo-HSCT	Tumor suppressor	Relapse, Survival	90	OS /DFS		Mutation of *TP53*,* MLL*,* RUNX1*	Allo-HSCT could remarkably overcome the adverse effect of low miR-425 expression. Thus, miR-425 might be considered as a predictive molecular marker to guide the treatment choice between allo-HSCT and chemotherapy
Gaoqi Zhang 2019 [4]	miR-500	qRT-PCR /In silico		74	32 females and 42 males	Allo-HSCT	Tumor suppressor	Relapse, Survival	140	OS	In low expression=:70.3%, In high expression = 67.6%	Mutation of *RUNX1*,* PHF6*,* TP53*,* KMT2A*	MiR-500 may be a suitable prognostic marker for patients with AML receiving alloHSCT
Huihui Zhang 2019 [25]	miR-363	qRT-PCR /In silico		162	Age: above or 60 years: 81 and under 60 years: 81. 71 females and 91 males	Allo-HSCT	Oncogene	Relapse, Survival	100	OS /DFS	Chemotherapy group: High miR-363: 93.3% and low miR-363: 62.2%. Allo-HSCT group: High miR-363: 63.9% and low miR-363: 47.2%	Mutation of *TP53*,* MLL-PTD*, and FLT3-ITD, and WBC count	MiR-363 expression may help identify patients in need of strategies to select the optimal therapy between chemotherapeutic and allo-HSCT regimens. AML patients with high miR-363 expression may be highly recommended for early allo-HSCT regimen
Jilei Zhang 2019 [26]	miR-425	qRT-PCR /In silico		162	Age: above or 60 years: 81 and under 60 years: 81 71 females and 91 males	72 Allo-HSCT, 90 Chemotherapy	Oncogene in allo-HSCT and tumor suppressor in chemotherapy group	Mortality, Relapse, Clinical outcome	120	OS /DFS	Chemotherapy group: High miR-425: 33.3% and low miR-425: 37.8%. Allo-HSCT group: High miR-425: 72.2% and low miR-425: 63.9%	Mutation of *TP53*, *DNMT3A*, and *IDH1/2*	MiR-425 is an independent favorable prognostic factor for younger AML patients undergoing chemotherapy, and its use may facilitate clinical decisionmaking in selecting treatment for AML patients
Jing-dong Zhou 2019 [16]	miR-195, miR-497, miR-193b	qRT-PCR, Bioinformatics analyses	BM	173	Age median: 61 (22–82) in low expression and 56 (18–88) in high expression. 81 females and 92 males	73 Allo and Auto-HSCT	Tumor suppressor	Relapse, Mutations, Clinical outcomes	120	OS /LFS		Age, WBC count, Karyotype risk, Treatment regimens, Mutation of *DNMT3A*,* RUNX1*, and *TP53*	Evaluation of microRNAs association with pathogenesis, treatment response and prognosis of AML patients (especially after HSCT)
Katja Seipel 2020 [22]	miR-181a-5p, miR-181b-5p, miR-20a5p	qRT-PCR	BM/ PB	54	Age median: 54 years. 27 females and 27 males	Auto-HSCT	Tumor suppressor	Relapse, Mutations, Clinical outcomes	10 years	OS/ PFS	68%		MiR-181a-5p are prognostic markers in AML patients treated with intensive induction chemotherapy and auto-SCT. The tumor suppressor miR-181a-5p may be a candidate miRNA mimic for therapeutic use
Lin Fu 2019 [5]	miR-338	qRT-PCR /In silico and Profiling		164	Age: above or 60 years: 81 and under 60 years: 83. 72 female sand 92 males	Allo-HSCT	Oncogene	Survival	120	OS /EFS	In chemotherapy group, high expression: 33.3% and low expression: 37.8%. In Allo-HSCT group, high expression: 64.9% and low expression: 70.3%	Mutation of *RUNX1* and FLT3-ITD	Upregulated miR-338 positively correlates with higher frequencies of complex karyotype, *RUNX1* mutation, and poor risk status, and finally shorter EFS in chemotherapy group. Allo‐HSCT could significantly overcome the negative effect of high miR‐338 expression
Mingshan Niu 2019 [18]	miR-25	qRT-PCR /In silico		162	Age: above or 60 years: 82 and under 60 years: 80. 71 females and 91 males	Allo-HSCT	Tumor suppressor in chemotherapy group	Relapse	90	OS/ EFS		Mutation of FLT3-ITD, *RUNX1*, *NPM1*, *DNMT3A*, *IDH1* and *IDH2* genes	MiR-25 levels are correlated with prognosis in AML independently of other powerful molecular markers. The expression of miR-25 may contribute to the selection of the optimal treatment regimen between chemotherapy and allo-HSCT for AML patients
Ning Hu 2019 [8]	miR-98	qRT-PCR /In silico		164	Age: above or 60 years: 81 and under 60 years: 83. 72 females and 92 males	Allo-HSCT	Tumor suppressor in chemotherapy group	Relapse	120	OS/ EFS	In chemotherapy group, High expression: 26.7% and low expression: 44.4%. In allo-HSCT group, High expression: 32.4%. and low expression: 32.4%		Patients with low miR-98 expression may benefit from allo-HSCT
Shi Jinlong 2015 [23]	miR-188-5p	qRT-PCR /In silico		200	Age median: 57 (21–88)	Allo and Auto-HSCT	Tumor suppressor	Relapse	80	OS/ EFS	63.7	Mutation of FLT3-ITD	Low miR-188-5p expression could be potentially used as favorably prognostic biomarkers independently or in a combined way in AML patients, whether they received HSCT or not
Ting-Juan Zhang 2020 [27]	miR-429, miR-29b	qRT-PCR /In silico		173	Age median: 57 (18–88). 47 and 45 males and 40 and 41 females in low and high. *DNMT3A* expression respectively.	Allo and Auto-HSCT			120	OS/ LFS		*DNMT3A* expression, Mutation of *FLT3*,* TP53*, and *RUNX1*	*DNMT3A* expression acted as a potential prognostic biomarker and may guide treatment choice between chemotherapy and HSCT in AML. miR-29b and miR-429 were identified as the predicted microRNAs that could target *DNMT3A* directly
Zhiheng Cheng 2017 [12]	miR-99a	qRT-PCR /In silico		74	Age: 18–72. 32 females and 42 males	Allo-HSCT	Tumor suppressor	Relapse	120	OS/EFS /LFS	67.5	Mutation of *TP53*,* PHF6*,* MLL-PTD*,* WT1* and *RUNX1*	High miR-99a expression could predict worse outcome in AML patients, even in those who underwent intensive post remission therapy such as allo-HSCT
Zhiheng Cheng 2021 [13]	miR-20b	qRT-PCR /In silico	PB	164	Age: 18–88. 72 females and 92 males	Allo-HSCT	Oncogene	Relapse	120	OS/ EFS	51.6	Mutation of *TP53* and FLT3-ITD	High miR-20b expression is a poor prognostic indicator for AML, but allo-HSCT may override its prognostic impact

BM: Bone marrow, PB: Peripheral blood

* Month

#### Patients with ALL

In a pioneering study conducted by Peter L. Pontoppidan et al., it was discovered that chemotherapy-induced toxic responses were accompanied by the differential regulation of miRs, which have opposing effects on immune regulation. During the period from day 0 to day + 28 following HSCT, miR-155 levels were found to be significantly elevated while miR-146a levels decreased in comparison to pre-conditioning levels. This indicates an inverse relationship between the two miRs. Additionally, the study revealed that a pro-inflammatory miRNA profile persisted during the first three weeks after transplantation, indicating that these miRs may have a role in regulating the inflammatory environment during immune reconstitution following HSCT [21]. In 2019, Xiao-Peng Tian et al. developed a five-miRNA-based classifier to predict the survival of patients with T-cell lymphoblastic lymphoma (T-LBL). By measuring the levels of five specific miRs (miR-513a, miR-21, miR-19b-3p, miR-638, and miR-26a-5p), patients were classified into either a low-risk group associated with a better prognosis or a high-risk group associated with a more favorable response to HSCT. Their study demonstrated that this classifier had strong predictive performance for adults with T-LBL. Moreover, the study revealed that HSCT could provide survival benefits for patients classified as high-risk by the five-miRNA classifier but not for those in the low-risk group [24]. In 2011, the methylation status of several miRs was identified by Vanesa Martin-Palanco et al. as a promising predictor of the ALL outcome after HSCT. They examined two groups based on the methylation profile: the CpG island methylator phenotype (CIMP) negative group (CIMP2), consisting of patients with 0–1 methylated gene, and the CIMP positive group (CIMP1), comprising patients with more than one methylated gene. They found that the CIMP2 group had longer DFS (disease-free survival) and OS, whereas the CIMP1 group had higher mortality and relapse rates. Importantly, the research team also demonstrated that the epigenetic regulation of miR-124a, one of the most frequently methylated microRNA families in their patients, mediated the increased expression of CDK6 and contributed to abnormal ALL cell proliferation in vitro and in vivo [17]. All the extracted data from ALL related studies is shown in Table 3.

**Table 3:** Extracted data from ALL related studies with miRs assessment in HSCT

Study	MiRs	Method	Sample type	No. Sample	Age and Sex of samples	HSCT type	Role of miRs	Follow up parameter	Follow up	Survival parameters	% Of relapse	Other measured parameters	Main finding or purpose
Vanesa Martın-Palanco 2010 [17]	miR-124-1, miR-124-3, miR-124-2, miR-129, miR − 193 A, miR − 375, miR − 203, miR − 34BC, miR − 9 − 1, miR − 9 − 2, miR − 9 − 3, miR − 132-2, miR-196B, miR − 10B	Methylation analysis	BM	32	12 females and 20 males	8 Auto-HSCT and 24 Allo-HSCT	Tumor suppressor	Relapse /Mortality	200 months	OS/DFS	Methylated: 20% Un-methylated: 64%		Effects of methylation status of several miRs on outcomes after HSCT in ALL patients
Xiao-Peng Tian 2019 [24]	miR-21-5p, miR-513a-5p, miR-19b-3p, miR-4742-3p, miR-1268a, miR-5690, miR-1207-5p, miR-3197, miR-4284, miR-1915-3p, miR-4748, miR-4780, miR-4665-3p, miR-4525, miR-1202, miR-3912, miR-720, miR-4766-5p, miR-4640-5p, miR-3935, miR-3618, miR-1275, miR-3616-5p, miR-4281, miR-4270, miR-4436-3p, miR-494, miR-1280, miR-3178, miR-3162-5p, miR-1260a, miR-5693, miR-149-3p, miR-638, miR-26a-5a	Array of miRs	Lymph nodes and thymus biopsy	517	Age range: 18–65 years 7 females and 13 males	HLA identical or haplo-identical HSCT	Oncogene Tumor suppressor	Complete remission Relapse, Mortality	42.8 weeks	OS and DFS	43.5% (225/517)	LDH level	MiRs expression associated with DFS. Five miRs can predict relapse after CR in adult T-LBL patients
Peter L Pontoppidan [21]	miR-146a, miR-155	qRT-PCR	PB	30	Age median: 18.5 (1–55). 10 females and 20 males	Allo-HSCT	-	-	28 days	-	-	-	Pro-inflammatory miRNA profile is sustained during the first three weeks after the transplantation, indicating that these miRNAs may play a role in the regulation of the inflammatory environment during immune reconstitution after HSCT

BM: Bone marrow, PB: Peripheral blood

#### Patients with MM

Table 4 depicts the precise extracted data about miRs’ effects on HSCT in MM-related studies. According to research conducted by Sung-Soo Park et al. in 2019, patients with MM who had high levels of circulating miR-193a-5p prior to autologous SCT had a better chance of avoiding early relapse and achieving favorable PFS. The study suggests that the amount of miR-193a-5p in the bloodstream could serve as a reliable predictor of the likelihood of early relapse as well as an independent factor in determining PFS. These findings could help improve the prognosis and treatment outcomes for MM patients undergoing ASCT [20]. MiRSNPs are introduced as prognostic markers for MM patients undergoing ASCT for the first time as two specific miRSNPs—rs3660 in KRT81 and rs11077 in XPO5—that have a significant prognostic impact after ASCT. Patients with the rs3660 C/C variant in KRT81 and the C/C or A/C variant in XPO5 rs11077 experience longer OS, while those with the C variant in KRT81 rs3660 have reduced protein levels and, because of that, decreased proliferation. The rs11077 SNP in XPO5 also showed increased PFS [14]. Circulatory microRNA expression has shown promise as a diagnostic and prognostic tool for MM. 14-microRNA signatures differentially express in MM patients’ serum and correlate with diagnosis and complete remission after ASCT. Patients with high levels of miR-19b or miR-331 have longer PFS after ASCT [7].

**Table 4:** Extracted data from MM related studies with miRs assessment in HSCT

Study	MiRs	Method	Sample type	No. Sample	Age and Sex of samples	HSCT type	Role of miRs	Follow up parameter	Follow up	Survival parameters	% OF relapse	Other measured parameters	Main finding or purpose
Alfons Navarro 2015 [7]	miR-16, miR-17, miR-18a, miR-19b, miR-20a, miR-20b, miR-24, miR-25, miR-27a, miR-30b, miR-152, miR-331, miR-374, miR-600	Array/ qRT-PCR	PB	33	Age median: 56 (25–66). 20 females and 10 males	Auto-HSCT	Tumor suppressor	Relapse	14 years	PFS	54%	Creatinine	Low expression of both miR-19b and miR-331 in combination was a marker of shorter PFS after HSCT. MicroRNA is a diagnostic and prognostic tool in MM
de Larrea 2012 [14]	MiRSNPs	SNP Analysis	BM/PB	192	Age median: 55 (26–67). 63 female sand 74 males	Auto-HSCT			4 years	OS and PFS		International staging system, Immunoglobulin isotype	MiRSNPs emerged as new promising markers for disease progression in cancer and specifically in multiple myeloma
Sung-Soo Park 2019 [20]	miR-193a-5p	qRT-PCR	PB	140		Auto-HSCT	Oncogene	Relapse	17 months	OS and PFS		ISS, cytogenetic risk, and circulating miR-331-3p expression	Expression of circulating miR-193a-5p before ASCT could be a prognostic biomarker for transplant-eligible MM

#### General group

There are studies in a variety of diseases undergoing HSCT evaluating the miRs impacts on HSCT-related processes and outcomes, as mentioned in Table 5. High expression of miR-125b, either in plasma or extracellular vesicles (EVs), decreases relapse-free survival (RFS) after ASCT [6]. Also, Mateusz Nowicki and colleagues have reported that miRs may play a role in hematopoietic recovery during the early post-transplant period and affect engraftment efficiency following HSCT. Higher levels of miR-15a, miR-16, miR-126, and miR-146a on day 0 of HSCT were associated with a longer time to engraftment. Furthermore, a positive correlation was found between the levels of miR-15a, miR-146a, and miR-223 measured on day + 7 and the time to engraftment [9]. MiR-155 expression in plasma and EVs was found to be a significant predictor of platelet/neutrophil engraftment after conditioning, along with other clinical factors such as chemotherapy courses after diagnosis, and these findings suggest that targeting miR-155 could be a promising therapeutic approach to improve outcomes for patients undergoing ASCT [28].

**Table 5:** Extracted data from studies with miRs assessment in HSCT in a variety of diseases

Study	MiRs	Method	Sample type	No. Sample	Age and Sex of samples	Disease type	HSCT type	Role of miRs	Follow up parameter	Follow up	Survival parameters	Other measured parameters	Main finding or purpose
Gretchen A 2017 [15]	miR-148a/b	qRT-PCR /In silico		7,327		AML, ALL, CML, MDS	Allo-HSCT		Relapse, Acute and chronic GVHD, Treatment-related mortality	10 years	OS /DFS		Recipient HLA-C Haplotypes and miR- 148a/b Binding Sites Have No Impact on Allo-HSCT Outcomes
Mateusz Nowicki 2016 [19]	miR-15a, miR-16, miR-126, miR-146a, miR-223	qRT-PCR	PB	51	Age median: 59 (36–69), 23 Females and 28 Males	MM, NHL, HD	Auto-HSCT	miRNA-233: Tumor suppressor	Engraftment	14 days		CD34^+^ cells	Slower Engraftment in Patients with High Expression of miR-15a, miR-16, miR-126, miR-146a, miR-223 Prior to Auto-HSCT and at Early Time after Transplantation
Mohamed L. Sorror 2018 [6]	miR-18a, miR-374b, miR-484, miR-374a, miR-106a + 17, miR-454, miR-520e, miR-590-3p, miR-590-5p, miR-148b, miR-191, miR-15b, miR-25, miR-142-3p, miR-29c, miR-148a, miR-1308, miR-424, miR-199b-5p	Nanostring Ncounter miR assay	PB	36	Age median: 49, 18 females and 18 males	AML, ALL	Allo-HSCT		Mortality, Acute GVHD	4 years	OS		MiRs-142-3p, -191, -424, -590-3p, -29c, and − 15b were overexpressed among high-risk patients relative to low-risk patients
Mohammad Rafiee 2021 [3]	miR-155	qRT-PCR	PB	50	Age median: 40, 25 females and 25 males	MM and Lymphomas	Auto-HSCT		Mobilization results, Engraftment	20 days		Chemotherapy courses after diagnosis, Total dose of G-CSF, CD34^+^ count per Kg, G-CSF dose after HSCs infusion, Disease type, Conditioning days	MiR-155 can predict the outcome of auto-HSCT, like engraftment

BM: Bone marrow, PB: Peripheral blood

In 2017, Gretchen A. Hoff et al. conducted a study that showed that neither HLA-C surface expression nor recipient HLA-C epitopes (C1, C2) are associated with outcomes in allo-HSCT. The study utilized a miR148a/b-binding SNP to determine HLA-C surface expression and found that this factor did not have a significant impact on allo-HSCT outcomes [15].

## Discussion

Extensive research has demonstrated that miRs can play both tumor suppressor and oncogene roles in different types of leukemia and can participate in various treatment processes such as differentiation, proliferation, and prognostication [4]. MiRs-based treatments are advancing rapidly. For example, miRs have been shown to be a significant prognostic factor in AML. Additionally, miRs play a critical role in maintaining hematopoietic stem cells and can bind to the 3’-untranslated region of their target mRNA and induce apoptosis or affect cell proliferation or differentiation. However, abnormal expression of miRs can lead to harmful outcomes [13, 18]. So, in this review, we focused on the effects of miRs on survival factors during the HSCT process. Additionally, we considered the effective treatment with the association of miRs between HSCT and chemotherapy.

Through this study, it has been determined that some mutations have indirect and direct effects on miR expression. For example, high expression of miR-425 exhibited high frequency of CBFß-MY11 and MLL-PTD in AML patients [26]. Additionally, it seems that the most high-expression miRs with better OS were tumor suppressor. However, some high-expression miRs like miR-98 and miR-25 had no specific effect on OS [4, 13, 18, 25]. But upregulation of miR-25 showed low frequency of FLT3-ITD mutation in AML [18]. Upon our knowledge from the search in studies, it is not completely determined the effect of miRs on leukemia-related mutations, however, the epigenetic influence of miRs (like miR-425) on the mutated genes expression is suggested [26].

In our evaluated studies, we have observed the significant role of certain miRs in predicting the prognosis of patients. The combination of high expression of let-7a-2-3p and low expression of miR-188-5p has shown promise in improving OS and EFS in patients with CN-AML, regardless of whether they undergo HSCT or not. Notably, this combination is associated with mutations in the *CEBPA* gene (CCAAT Enhancer Binding Protein Alpha), which serves as a favorable prognostic biomarker in CN-AML patients [23].

Conversely, elevated expression of miR-500 has been linked to lower OS and EFS in AML patients undergoing allogeneic HSCT. This high expression of miR-500 is associated with mutations in the *PHF6* gene, a tumor suppressor gene. Consequently, the combination of high mir-500 expression and a *PHF6* gene mutation correlates with a poor prognosis [4]. Furthermore, the presence of high miR-363 expression in chemotherapy-treated groups has been associated with longer OS and EFS. However, in the context of allo-HSCT, there are no significant differences observed between the high- and low-expression groups of miR-363. Nevertheless, allo-HSCT can counteract the negative impact of high miR-363 expression. Regarding the regulatory role of miR-363, its expression has been found to significantly affect the MLLT3 regulation, leading to increased *AF9* expression. Conversely, reduced levels of miR-363 have been linked to *NPM1* and CBFß-MY11. These findings underscore the importance of miR-363 in regulating these pathways [25]. High levels of circulating miR-193a-5p prior to ASCT in MM patients have demonstrated a better likelihood of avoiding early relapse and achieving favorable PFS [20]. These studies are compelling examples highlighting the importance of monitoring the impact of miRs in various clinical contexts.

## Future perspectives

Finally, according to the findings of this systematic review, the following recommendations and future perspectives about this domain can be useful in enhancing the understanding and clinical application of microRNAs in the field of HSCT. Conducting comprehensive functional studies is crucial to elucidating the valuable roles and mechanisms of action of the identified miRs and their combinations. Future studies should encompass various cellular and molecular processes relevant to HSCT, such as stem cell maintenance, engraftment, immune reconstitution, disease progression, and so on. Also, future studies should be a combination of different study design models (the findings should not only be in-silico and human samples should also be examined) and conduct multivariate analyses about the different parameters relevant to the role of miRs in different stages of the disease (such as before and after chemotherapy and HSCT), patients with different cytogenetics, and MRD, which can be useful regarding the reliability of the results. Second, prospective cohort studies with larger sample sizes should be done to validate the prognostic and predictive value of the identified miRs. Cohort studies should include comprehensive clinical data, detailed molecular profiling, and a suitable longitudinal follow-up period to establish robust associations between miR expression patterns and HSCT outcomes (such as OS, DFS, relapse rates, treatment-related complications, and so on). Third, integrative Multi-Omics approaches can be valuable. Integrating miR profiling with other omics data, such as genomics, transcriptomics, and proteomics, can provide a more comprehensive understanding of the molecular landscape underlying HSCT outcomes. This integrated approach may reveal previously unrecognized interactions between miRs and other regulatory elements, such as coding genes, transcription factors, and signaling pathways, and finally illustrate the complex regulatory networks involved in the pathogenesis of disease and treatment responses, such as HSCT. Fourth, regarding the findings of functional studies, efforts can be directed towards developing miR-based therapeutic strategies for HSCT (development of miR-based therapeutic strategies). These strategies may encompass miR mimics or inhibitors, as well as targeted delivery systems to enhance specificity and efficacy. In the first step, preclinical studies evaluating the safety, toxicity, and efficacy of these therapeutic approaches should be done, finally paving the way for future clinical trials. And the fifth suggestion is miRs’ biomarker development and clinical implementation. Translating the identified miRs as clinically prognostic and diagnostically validated biomarkers is a crucial step towards personalized medicine and improved risk stratification for HSCT patients. Robust validation studies should be done to make standardized protocols for miR quantification and analysis and to ensure the reproducibility and reliability of miRs in clinical translation. Additionally, integrating miR biomarkers into existing clinical decision-making algorithms and risk assessment models can provide more informed treatment choices and optimize patient outcomes. Finally, by implementing these recommendations and future perspectives, the scientific community can be using the full potential of miRs for better understanding and clinical management of HSCT. This concerted effort will contribute to the development of personalized medicine treatments, the determination of risk stratification, and ultimately, improved outcomes for patients.

## Limitations

Our study, like many other systematic reviews, has certain limitations that should be acknowledged. One limitation is that we were unable to access or obtain the full text of all articles that were identified based on their titles. This could be due to the unavailability of the full text for purchase or restricted access based on geographical limitations. Second, some of the articles we reviewed did not provide the specific information we were seeking, even though they were related to miRs, and their focus did not align with our research topic of interest. The third challenge we encountered was the heterogeneity of the articles included in our review. We observed diverse results, approaches, and emphases across the studies. Some articles primarily focused on OS and EFS, while others emphasized relapse rates or engraftment, and some studies included in our review did not have suitable follow-up durations, which may not show the full extent of HSCT-related outcomes and their long-term effects. Fourth, working with cohort articles was challenging due to the lack of information on certain aspects we wished to investigate, such as inadequate details on methods or insufficient data regarding miRNAs acting as oncogenes or tumor suppressors. Fifth, the techniques and methodologies that had been used for miR profiling and quantification varied across studies, potentially leading to discrepancies in the sensitivity, specificity, and reproducibility of the results. In the sixth, a notable proportion of the included studies had relatively small sample sizes, which can potentially limit the statistical power and generalizability of their findings, and the seventh item is about potential publication bias. Studies with negative or non-significant findings in the in the literature lead to potential publication bias.

## Conclusion

This systematic review is the first of its kind to explore the impact of miRs on patients undergoing HSCT. The findings of this study highlight the immense potential of miRs in predicting the effectiveness of HSCT and emphasize the importance of understanding their role in improving patient assessments. However, further research employing advanced techniques and refined approaches is necessary to validate and verify our findings, ultimately enhancing our understanding of how miRs can contribute to a more accurate assessment of patients’ conditions and optimize treatment outcomes. In the future, the strategic targeting of miRs using improved drugs or antibodies may provide promising results, such as increased overall survival rates, reduced relapse rates, and enhanced engraftments. These advancements have the potential to shape better treatment protocols for patients undergoing HSCT.

## Data Availability

All data generated or analyzed during this study are included in this published article.

## Abbreviations

MiRs: MicroRNs
HSCT: Hematopoietic stem cell transplantation
Allo-HSCT: Allogeneic hematopoietic stem cell transplantation
Auto-HSCT or ASCT: Autologous hematopoietic stem cell transplantation
T-LBL: T-cell lymphoblastic lymphoma
OS: Overall survival
EFS: Event-free survival
PFS: Progression-free survival
RFS: Relapse-free survival
